# Changes in lipid profiles during and after (neo)adjuvant chemotherapy in women with early-stage breast cancer: A retrospective study

**DOI:** 10.1371/journal.pone.0221866

**Published:** 2019-08-29

**Authors:** Wei Tian, Yihan Yao, Guocai Fan, Yunxiang Zhou, Miaowei Wu, Dong Xu, Yongchuan Deng

**Affiliations:** 1 Department of Surgical Oncology, The Second Affiliated Hospital, School of Medicine, Zhejiang University, Hangzhou, Zhejiang, China; 2 Department of Breast Surgery, The People’s Hospital of Suichang County, Lishui, Zhejiang, China; Tabriz University of Medical Sciences, ISLAMIC REPUBLIC OF IRAN

## Abstract

Many different treatments may affect the serum lipid profiles of breast cancer patients. This study analyzed serum lipid levels at different periods during treatment to observe the changes in lipid profiles during and after chemotherapy and to compare the different effects of different chemotherapy regimens on serum lipid profiles. A total of 805 patients were included in this study. We measured the lipid profiles of patients who received surgery without chemotherapy prior to the operation and at 3, 6 and 12 months after operation. In addition, in patients who underwent chemotherapy, the lipid profiles were measured prior to chemotherapy, prior to the last cycle of chemotherapy and 6 months after chemotherapy. Lipid profile measurements included total cholesterol (TC), triglycerides (TG), low-density lipoprotein cholesterol (LDL-C), high-density lipoprotein cholesterol (HDL-C), homocysteine (HCY), and uric acid (UA). (Neo)Adjuvant chemotherapy exerted an adverse temporary effect on lipid levels (manifested as increased TG and LDL-C levels, and decreased HDL-C levels, particularly in the adjuvant chemotherapy group) during the chemotherapy periods. However, this influence was not sustained, as the lipid profiles levels were generally restored to baseline levels 6 months after chemotherapy completion. Different age groups showed different changes in lipid levels that were influenced by chemotherapy. The younger group (20–40 years old) showed a greater increase in TC and LDL-C levels during chemotherapy than the 41-65-year-old group. Chemotherapy exerts an adverse temporary effect, and the effects of different regimens on lipid levels are similar. Furthermore, lipid profiles in younger women may be more sensitive to chemotherapy.

## Introduction

Breast cancer is the most common malignant tumor and one of the main causes of cancer-related death in women[1–3]. Survival rates of breast cancer patients are increasing because of normative systemic treatment, including chemotherapy, targeted therapy, endocrine therapy, radiotherapy and various supportive treatments. Approximately 90% of breast cancer patients survive for at least 5 years after their initial diagnosis[4]. Some breast cancer patients eventually die of diseases other than breast cancer, especially cardiovascular disease (CVD)[5]. CVD and breast cancer share some predisposing risk factors, such as diet, obesity, and a sedentary lifestyle[6, 7]. On the other hand, long-term survivors may develop latent cardiac effects secondary to the cancer treatment (including chemotherapy, radiotherapy and targeted therapy). The above risk factors increase the mortality of CVD in older postmenopausal women (≥66 years old) who are long-term survivors (≥5 years). For these women, CVD exceeds breast cancer as the leading cause of death at 10 years after diagnosis[8]. Accordingly, we must focus on detecting the potential risk of CVD throughout process of diagnosis and management in these breast cancer patients.

For early-stage breast cancer patients, chemotherapy is an essential treatment to improve disease-free and overall survival. However, chemotherapy is associated with long-term side effects, including CVD. CVD (such as heart failure, myocardial ischemia, hypertension) has emerged as a common complication after chemotherapy[9, 10], not only due to the direct cardiotoxicity of chemotherapy drugs but also due to the impact on serum lipid levels. Anthracyclines induce irreversible cardiotoxicity, depending on its cumulative dose[11–13]. Regarding serum lipid levels, dyslipidemia (such as changes in high low-density lipoprotein cholesterol and low high-density lipoprotein cholesterol and hypertriglyceridemia) is a primary and major risk factor for atherosclerotic cardiovascular disease (ASCVD)[14, 15]. High serum cholesterol levels, especially high LDL-C, is causal and an independent risk factor for ASCVD[16, 17]. A low HDL-C level acts synergistically with other lipid risk factors to increase the ASCVD risk. Some studies have reported the effects of endocrine therapy and radiotherapy on serum lipid levels in breast cancer patients, including our group[18–21]. However, few studies have investigated the effect of chemotherapy on serum lipid levels. Therefore, a discussion of the effect of chemotherapy on serum lipid level during and after the completion of chemotherapy is important. Furthermore, researchers have not clearly determined whether chemotherapy treatments affect lipid profiles differently. This retrospective study analyzed the changes in lipid profiles during and after chemotherapy, and compared the different effects of different chemotherapy regimens on serum lipid profiles.

## Material and methods

### Study participants and design

Overall, we retrospectively analyzed breast cancer patients who underwent surgery, were treated with or without (neo)adjuvant therapy and attended follow-up visits at the Second Affiliated Hospital of Zhejiang University from January 1, 2012, to December 31, 2017. We included the patients on November 4, 2018, and the dates of data collection ranged from November 4, 2018 to November 15, 2018. Exclusion criteria included patients older than 65 years, patients who had dyslipidemia or took drugs affecting lipid levels, patients diagnosed with other malignant tumors or breast cancer recurrence or metastasis, patients with certain cardiovascular diseases (including coronary artery disease and stroke), diabetes mellitus or who took drugs affecting blood glucose levels.

All included patients accepted radical surgery. They were administered neoadjuvant or adjuvant chemotherapy according to clinicopathological manifestations and molecular subtypes. Hormone receptor (HR)-positive patients received adjuvant endocrine therapy, and HER-2-positive patients received trastuzumab concurrent with chemotherapy.

Patients were stratified into the following groups based on different treatments: a surgery group (received surgery but not chemotherapy), adjuvant chemotherapy group (received adjuvant chemotherapy after surgery) and neoadjuvant chemotherapy group (received neoadjuvant chemotherapy before surgery). For chemotherapy groups, participants were further divided into an anthracycline chemotherapy group (EC regimen), taxane chemotherapy group (TC, TCH or PH) and anthracycline plus taxane group (TEC or EC followed by P/T(H)). Medicine and disease histories, pathological results, immunohistochemistry results, tumor sizes, surgery approach and laboratory test results were obtained for each participant at baseline. The blood lipid levels were collected and analyzed from the database of the Second Affiliated Hospital of Zhejiang University. The levels of total cholesterol (TC), triglycerides (TG), low-density lipoprotein cholesterol (LDL-C), high-density lipoprotein cholesterol (HDL-C), homocysteine (HCY), and uric acid (UA), as well as the body mass index (BMI), were analyzed 4 times in the surgery group (prior to the operation and 3, 6 and 12 months after the operation) and 3 times in the chemotherapy groups (prior to chemotherapy, prior to the last cycle of chemotherapy and 6 months after chemotherapy).

Researchers had advised patients presenting substantial increases in blood lipid levels to seek a clinical consultation at the cardiovascular department during the follow-up period.

This study has been approved by the Ethics Committee of the Second Affiliated Hospital of Zhejiang University School of Medicine. In addition, the Ethics Committee waived the requirement for consent.

### Plasma lipid measurements

LDL-C, HDL-C, HCY and UA levels were detected using direct methods, and the glycerol-phosphoric acid oxidase peroxidase method was used to measure the serum TG level. The cholesterol oxidase method was used to determine the serum TC level, as described previously[19].

### Statistical analyses

Statistical analyses were performed using SPSS software version 20.0. Statistical descriptions, including means, standard deviations (SD), ranges and percentages, were used to characterize the eligible participants. Correlation test was used to investigate the correlations of different lipid measurements. A repeated measurement analysis of variance (RMANOVA) was performed to compare the differences in lipid levels at different time points before, during and after the interventions. The main effects included in the RMANOVA was the time. In addition, analysis of covariance was used to analyze the differences in changes in lipid levels between patients receiving different chemotherapy regimens and to analyze the differences in lipid levels between two year groups. As BMI levels also affected the lipid profiles, we also included BMI as a concomitant confounding variable in the analysis of covariance. P<0.05 was considered statistically significant. The least significant difference (LSD)-t test was used for multiple comparisons.

## Results

### Study population

**Fig 1:**
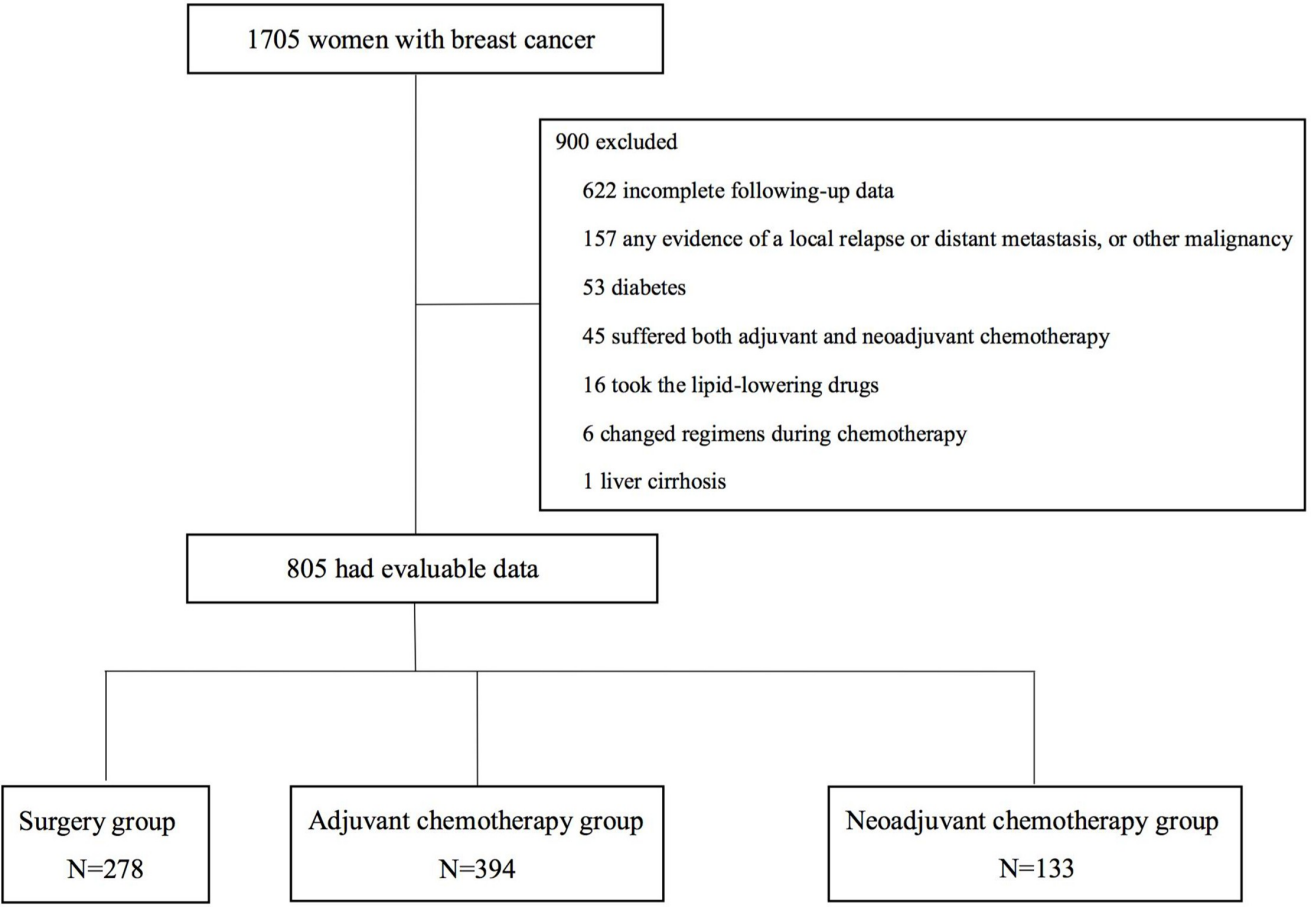
Diagram of included and excluded participants in this lipid analysis.

A total of 1705 patients were enrolled in this study, of which 805 had evaluable data and satisfied the inclusion criteria (Fig 1). Of the 805 participants, patients were divided into 3 groups based on the different treatments received: a surgery group (n = 278), adjuvant chemotherapy group (n = 394) and neoadjuvant chemotherapy group (n = 133). The baseline characteristics of 3 groups of patients are shown in Table 1. Patients’ demographics and baseline characteristics were balanced among three groups.

**Table 1:** Baseline characteristics of patients.

Characteristic	Surgery (n = 278)	Adjuvant chemotherapy (n = 394)	Neoadjuvant chemotherapy (n = 133)
**Age, years** **Mean (range)**	48.8 (23–65)	48.9 (26–65)	49.3 (26–65)
**BMI, kg/m**^**2**^ **Mean (SD)**	22.55 (3.00)	22.60 (2.78)	23.27 (3.14)
**Type of surgery**			
**Breast-conserving**	103 (37.0%)	152 (38.6%)	25 (18.8%)
**Mastectomy**	175 (63.0%)	242 (61.4%)	105 (78.9%)
**Unknown**	0	0	3* (2.3%)
**Depth of tumor invasion (T)**			
** Tis**	144 (51.8%)	6 (1.5%)	0
** T1**	109 (39.2%)	260 (66.0%)	61 (45.9%)
** T2**	21 (7.6%)	118 (30.0%)	65 (48.9%)
** T3-T4**	1 (0.4%)	4 (1.0%)	7 (5.2%)
**Unknown**	3^#^ (1.0%)	6^#^ (1.5%)	0
**Lymph node metastasis (N)**			
**N0**	267 (96.0%)	273 (69.3%)	21 (15.8%)
**N1**	11 (4.0%)	100 (25.4%)	94 (70.7%)
**N2-N3**	0	21 (5.3%)	18 (13.5%)
**ER status**			
**Negative**	52 (18.7%)	107 (27.2%)	53 (39.8%)
**Positive**	225 (80.9%)	279 (70.8%)	77 (57.9%)
**Unknown**	1 (0.4%)	8 (2.0%)	3 (2.3%)
**PR status**			
**Negative**	51 (18.3%)	135 (34.3%)	69 (51.9%)
**Positive**	226 (81.3%)	252 (63.9%)	61 (45.9%)
**Unknown**	1 (0.4%)	7 (1.8%)	3 (2.2%)
**HER-2 status**			
**Negative**	189 (68.0%)	258 (65.5%)	85 (63.9%)
**Positive**	61 (21.9%)	134 (34.0%)	47 (35.3%)
**Unknown**	28^&^ (10.1%)	2^#^ (0.5%)	1^#^ (0.8%)
**Ki-67 status**			
**<20%**	167 (60.1%)	112 (28.4%)	58 (43.6%)
**≥20%**	94 (33.8%)	260 (66.0%)	64 (48.1%)
**Unknown**	17 (6.1%)	22 (5.6%)	11 (8.3%)
**Endocrine therapy**			
**Yes**	228 (82.0%)	285 (72.3%)	82 (61.7%)
**No**	50 (18.0%)	109 (27.7%)	51 (38.3%)
**Radiation therapy**			
**Yes**	101 (36.3%)	213 (54.1%)	107 (80.5%)
**No**	177 (63.7%)	181 (45.9%)	26 (19.5%)

BMI: Body mass index; SD: Standard deviation; ER: Estrogen receptor; PR: Progesterone receptor

*: Had not received the operation for breast cancer at the Second Affiliated Hospital of Zhejiang University after neoadjuvant chemotherapy.

^#^: No information was available.

^&^: No further FISH test in many patients with intraductal carcinoma.

### Lipid profiles of the surgery group

The mean lipid levels, standard deviations and the comparisons between different time points in the surgery group are shown in Table 2. Clearly, the TG and UA levels increased significantly after surgery (3 and 6 months after the operation for TG levels and 3, 6, and 12 months after the operation for UA levels), while TC, LDL-C, HDL-C and HCY levels decreased significantly after surgery (3, 6, and 12 months after operation for TC and LDL-C levels, 3 and 6 months after the operation for HDL-C levels, 6 months after operation for HCY levels). BMI levels were not significantly altered from 3–12 months after surgery.

**Table 2:** Changes in the levels of 7 parameters in the surgery group.

Surgery group		Preoperation	3 months postoperation	6 months postoperation	12 months postoperation
**TC**	**Mean±SD**	5.04±0.98	4.80±0.96*	4.85±0.99*	4.77±0.96*
**TG**	**Mean±SD**	1.43±1.13	1.77±1.27*	1.72±1.14*	1.65±1.11*
**LDL-C**	**Mean±SD**	2.68±0.73	2.56±0.76*	2.57±0.78*	2.50±0.77*
**HDL-C**	**Mean±SD**	1.40±0.34	1.33±0.34*	1.36±0.32*	1.40±0.34
**HCY**	**Mean±SD**	10.42±6.51	10.04±5.64	9.82±4.79*	10.34±4.14
**UA**	**Mean±SD**	262.41±55.58	285.73±65.60*	287.70±59.49*	298.11±59.62*
**BMI**	**Mean±SD**	22.57±2.96	22.58±2.89	22.59±2.91	22.52±2.90

TC: total cholesterol (mmol/L); TG: triglycerides (mmol/L); LDL-C: low-density lipoprotein cholesterol (mmol/L); HDL-C: high-density lipoprotein cholesterol (mmol/L); HCY: homocysteine (μmol/L); UA: uric acid (μmol/L); BMI: body mass index; SD: standard deviation

* P<0.05 compared with the baseline level (preoperation level)

### Lipid profiles of the adjuvant chemotherapy and neoadjuvant chemotherapy groups

The mean lipid levels, standard deviations and the comparisons between different time points in the adjuvant chemotherapy and neoadjuvant chemotherapy groups are presented in Table 3 and Table 4, respectively. The result of correlation test showed that these lipid measurements were correlated to each other except HCY levels. For the adjuvant chemotherapy group, the TC, TG and LDL-C levels and BMI increased significantly during chemotherapy, while HDL-C and HCY levels decreased significantly during chemotherapy. Furthermore, when the levels of these lipids were analyzed 6 months after chemotherapy, the TC and LDL-C levels decreased and were even lower than the prechemotherapy levels. In contrast, HDL-C and HCY levels increased after chemotherapy completion and were restored to baseline levels. In addition, TG levels and BMI were still higher than the prechemotherapy levels. For the neoadjuvant chemotherapy group, the TG and LDL-C level increased significantly during chemotherapy, showing a similar trend to the adjuvant chemotherapy group. However, the increase in TC and UA levels during chemotherapy was not significant, and at 6 months after chemotherapy completion, TG and UA levels were significantly higher than the baseline values.

**Table 3:** Changes in the levels of 7 parameters in the adjuvant chemotherapy group.

Adjuvant chemotherapy group		Prechemotherapy	Prior to the last cycle of chemotherapy	6 m after chemotherapy
**TC**	**Mean±SD**	5.10±1.09	5.20±0.98*	4.95±1.02*^#^
**TG**	**Mean±SD**	1.64±0.96	2.12±1.37*	1.97±1.69*
**LDL-C**	**Mean±SD**	2.86±0.76	2.97±0.78*	2.67±0.76*^#^
**HDL-C**	**Mean±SD**	1.34±0.32	1.21±0.31*	1.35±0.34^#^
**HCY**	**Mean±SD**	9.43±2.38	9.00±2.44*	9.58±2.48^#^
**UA**	**Mean±SD**	270.05±55.12	272.80±61.22	291.51±58.70*^#^
**BMI**	**Mean±SD**	22.44±2.71	22.82±2.86*	22.69±2.82*

TC: total cholesterol (mmol/L); TG: triglycerides (mmol/L); LDL-C: low-density lipoprotein cholesterol (mmol/L); HDL-C: high-density lipoprotein cholesterol (mmol/L); HCY: homocysteine (μmol/L); UA: uric acid (μmol/L); BMI: body mass index; 6 m: 6 months; SD: standard deviation

* P<0.05 compared with the baseline level (prechemotherapy level)

^#^ P<0.05 compared with the level measured prior to the last cycle of chemotherapy

**Table 4:** Changes in the levels of 7 parameters in the neoadjuvant chemotherapy group.

Neoadjuvant chemotherapy group		Prechemotherapy	Prior to the last cycle of chemotherapy	6 m after chemotherapy
**TC**	**Mean±SD**	5.14±1.08	5.34±1.13	5.19±1.20
**TG**	**Mean±SD**	1.42±0.78	1.97±1.02*	1.86±0.83*
**LDL-C**	**Mean±SD**	2.82±0.78	3.14±0.82*	2.92±0.88
**HDL-C**	**Mean±SD**	1.33±0.31	1.17±0.24	1.33±0.28
**HCY**	**Mean±SD**	11.44±9.46	9.65±2.57	10.56±2.45
**UA**	**Mean±SD**	270.78±59.44	274.28±62.22	301.06±60.66*
**BMI**	**Mean±SD**	23.07±3.04	23.16±2.87	23.22±2.88

TC: total cholesterol (mmol/L); TG: triglycerides (mmol/L); LDL-C: low-density lipoprotein cholesterol (mmol/L); HDL-C: high-density lipoprotein cholesterol (mmol/L); HCY: homocysteine (μmol/L); UA: uric acid (μmol/L); BMI: body mass index; 6 m: 6 months; SD: standard deviation

* P<0.05 compared with the baseline level (prechemotherapy level)

These data show the lipid profiles of the neoadjuvant and adjuvant chemotherapy groups. We subsequently analyzed the lipid profiles in groups stratified according to the use of different chemotherapeutic regimens. However, because of the limited number of patients in the anthracycline chemotherapy regimen subgroup of the neoadjuvant chemotherapy group (n = 2), the participants in the neoadjuvant chemotherapy group were not further analyzed according to the use of different chemotherapeutic regimens.

### Lipid profiles of patients receiving different chemotherapy regimens in the adjuvant chemotherapy group

Comparisons of lipid profiles between the 3 different adjuvant chemotherapy regimen groups are presented in Table 5.

**Table 5:** Levels of 7 parameters in patients receiving different chemotherapy regimens throughout chemotherapy.

Parameter	Regimen	Prechemotherapy	Prior to the last cycle of chemotherapy	6 m after chemotherapy
		Mean±SD	Mean±SD	Mean±SD
**TC**	Anthracycline	5.09±1.09	5.25±1.15	4.93±0.80
	Taxane	5.10±1.09	5.18±0.89	4.87±1.07*
	Anthracycline+taxane	5.10±1.09	5.19±1.02	5.05±1.05
**TG**	Anthracycline	1.68±0.81	2.14±1.52*	2.13±2.05
	Taxane	1.60±1.00	2.04±1.13*	2.00±1.91*
	Anthracycline+taxane	1.68±0.98	2.21±1.56*	1.85±1.12*
**LDL-C**	Anthracycline	2.89±0.79	3.05±0.91	2.62±0.64*
	Taxane	2.84±0.74	2.92±0.69	2.59±0.76*^&^
	Anthracycline+taxane	2.87±0.78	3.01±0.83*	2.78±0.80
**HDL-C**	Anthracycline	1.39±0.41	1.26±0.29*^&^	1.38±0.36
	Taxane	1.35±0.32	1.24±0.34*^&^	1.34±0.35
	Anthracycline+taxane	1.32±0.27	1.16±0.27*	1.34±0.30
**HCY**	Anthracycline	9.11±2.38	9.39±2.86	8.81±2.05
	Taxane	9.60±2.35	9.10±2.23*	9.84±2.13^#^
	Anthracycline+taxane	9.36±2.42	8.72±2.51*	9.59±2.96
**UA**	Anthracycline	283.41±60.69	296.22±77.25*	293.67±54.52
	Taxane	266.48±51.59	268.12±58.50^#^	292.36±57.36*
	Anthracycline+taxane	268.77±56.57	268.52±54.62^#^	289.47±62.50*
**BMI**	Anthracycline	22.27±2.55	22.37±2.67	22.43±2.74
	Taxane	22.46±2.86	22.92±2.92*^#^	22.66±2.89
	Anthracycline+taxane	22.50±2.60	22.89±2.88*	22.85±2.77*

TC: total cholesterol (mmol/L); TG: triglycerides (mmol/L); LDL-C: low-density lipoprotein cholesterol (mmol/L); HDL-C: high-density lipoprotein cholesterol (mmol/L); HCY: homocysteine (μmol/L); UA: uric acid (μmol/L); BMI: body mass index; 6 m: 6 months; SD: standard deviation

*: P<0.05 compared with baseline levels (prechemotherapy levels)

^#^: P<0.05 compared with the group receiving the anthracycline chemotherapy regimen

^&^: P<0.05 compared with the group receiving the anthracycline + taxane chemotherapy regimen

TC levels were slightly increased before the last cycles of chemotherapy compared with the prechemotherapy levels in all three regimen subgroups. At 6 months postchemotherapy, TC levels returned to baseline levels, except in the taxane group, in which the levels were significantly decreased compared with baseline levels (P<0.05). The three different chemotherapy regimens showed no significant difference in the change in TC levels.

TG levels increased significantly during chemotherapy in all 3 chemotherapy regimen groups. At 6 months after chemotherapy completion, the TG levels were slightly decreased but were still higher than baseline levels (prechemotherapy level) in all three groups, particularly in the groups receiving the taxane and anthracycline combined with taxane chemotherapy regimens (P<0.05). Furthermore, a significant difference in changes in TG levels was not observed between the groups receiving the three different chemotherapy regimens during chemotherapy and after chemotherapy completion.

The LDL-C levels were significantly increased during chemotherapy in the anthracycline plus taxane group (P<0.05), but were restored to baseline levels 6 months after chemotherapy completion. In the anthracycline group and taxane group, the LDL-C levels were slightly increased (but not significantly) during chemotherapy and decreased thereafter. Significantly lower LDL-C levels were observed 6 months after chemotherapy completion than at baseline in these two groups (P<0.05). Similarly, a significant difference in the change in LDL-C levels was not observed between patients receiving the three chemotherapy regimens during chemotherapy; however, at 6 months after chemotherapy completion, the group treated with the combination of anthracycline and taxane presented a higher LDL-C level than the taxane group (P<0.05).

The HDL-C levels decreased significantly during chemotherapy in all 3 regimen groups, and a more substantial decrease was observed in the group treated with the combination of anthracycline and taxane compared with the other two groups. At 6 months after chemotherapy completion, the levels were nearly restored to baseline levels. Furthermore, the effect size (partial eta squared) of chemotherapy regimens on HDL-C levels was 0.02. It means that although HDL-C levels showed a significant decrease in the group treated with the combination of anthracycline and taxane, the effect was small.

The HCY levels decreased significantly in the taxane group and anthracycline plus taxane group during chemotherapy (P<0.05) and were restored to baseline levels 6 months after chemotherapy completion. However, a significant decrease was not observed in the anthracycline group. In addition, a significant difference in HCY levels was not observed between the different chemotherapy regimens.

The UA level was only significantly increased in the anthracycline chemotherapy regimen group during chemotherapy. During chemotherapy, the UA level in anthracycline group was significantly higher than the taxane group and anthracycline plus taxane group. Furthermore, at 6 months after chemotherapy completion, the UA level in the anthracycline group was restored to the baseline level, while the levels in the other 2 groups were higher than baseline levels.

The BMI increased significantly in the taxane group and anthracycline plus taxane group during chemotherapy, while the BMI of the anthracycline group was not significantly increased throughout and after chemotherapy. In addition, the BMI of the taxane group was restored to the baseline level 6 months after chemotherapy completion, while the BMI of the anthracycline plus taxane group did not change at this time point.

### Lipid profiles of the adjuvant chemotherapy group after stratification into different age groups

Considering the differences in lipid metabolism in women of different ages, the adjuvant chemotherapy group was further stratified into a 20-40-year-old group and a 41-65-year-old group. The mean lipid levels, standard deviations and comparisons at different time points are presented in Table 6.

**Table 6:** Levels of 7 parameters in different age groups throughout chemotherapy.

Parameter	Group	Prechemotherapy	Prior to the last cycle of chemotherapy		6 m after chemotherapy	
		Mean±SD	Mean±SD	Change	Mean±SD	Change
**TC**	20–40	4.63±1.01	5.01±0.96	0.39	4.48±0.79	-0.03
	41–65	5.26±1.11*	5.25±0.96	-0.01^#^	5.04±1.04	-0.18
**TG**	20–40	1.26±0.63	1.93±1.48	0.67	1.68±1.92	0.47
	41–65	1.75±0.98*	2.14±1.25	0.39	2.02±1.63	0.30
**LDL-C**	20–40	2.50±0.77	2.79±0.77	0.29	2.24±0.63	-0.19
	41–65	2.97±0.77*	3.03±0.76	0.06^#^	2.75±0.76	-0.20
**HDL-C**	20–40	1.35±0.28	1.27±0.36	-0.08	1.41±0.38	0.08
	41–65	1.35±0.33	1.20±0.29	-0.15	1.33±0.33	-0.01
**HCY**	20–40	8.54±2.26	8.02±1.56	-0.55	8.49±1.66	-0.23
	41–65	9.69±2.32*	9.43±3.02	-0.37	9.79±2.56	0.22
**UA**	20–40	260.97±55.73	264.31±54.21	3.34	278.72±40.85	20.96
	41–65	273.85±55.97	273.98±60.85	0.12	294.02±61.36	21.55
**BMI**	20–40	21.36±2.78	21.87±3.02	0.50	21.69±2.78	0.45
	41–65	22.70±2.67*	23.04±2.80	0.34	22.89±2.79	0.21

Change indicates the absolute change from baseline levels. TC: total cholesterol (mmol/L); TG: triglycerides (mmol/L); LDL-C: low-density lipoprotein cholesterol (mmol/L); HDL-C: high-density lipoprotein cholesterol (mmol/L); HCY: homocysteine (μmol/L); UA: uric acid (μmol/L); BMI: body mass index; 6 m: 6 months; SD: standard deviation

*: P<0.05 compared with the level of the same parameter in the 20-40-year-old group at a specific time point

^#^: P<0.05 compared with the change in the level of the same parameter in the 20-40-year-old group at a specific time point

When we compared baseline (prechemotherapy) levels between the different age groups, the baseline TC, TG, LDL-C, and HCY levels and BMI of the 41-65-year-old group were higher than the 20-40-year-old group (P<0.05), while HDL-C and UA levels were not significantly different prior to chemotherapy.

In a comparison of the extent of the changes in the levels of the 7 parameters in different age groups during chemotherapy and after chemotherapy completion, TC and LDL-C levels showed a greater increase in the 20-40-year-old group during chemotherapy (P<0.05). Furthermore, the levels of both levels were restored to baseline levels at 6 months after chemotherapy completion.

## Discussion

Based on the results from the present study, almost all serum lipid levels exhibited significant changes after surgery in the surgery group, with stable BMI. Surgery altered the serum lipid profiles. The changes in nutritional status, dietary structure and physical exercise, and the removal of a large quantity of adipose tissue during surgery may explain the changes in the lipid profiles of breast cancer patients after surgery. However, the detailed explanations for this phenomenon remain unclear and require further study.

In the adjuvant chemotherapy group, significant changes in the levels of some components of the lipid profile were observed during chemotherapy (TC, TG and LDL-C levels increased, while HDL-C and HCY levels decreased), the changes in UA levels after chemotherapy were not significant. However, at 6 months after chemotherapy completion, the HDL-C and HCY levels were restored to the baseline level, the TC and LDL-C level decreased to an even lower value than before chemotherapy, while the TG and UA levels were significantly higher than the baseline level. Changes observed during chemotherapy are partially consistent with some previous studies. Li, Xin and coworkers conducted a retrospective study to analyze the levels of lipids and lipoproteins before and after chemotherapy, and observed significantly higher TC, TG, and LDL-C levels in patients after chemotherapy than before chemotherapy, while the HDL-C level showed the opposite trend[22]. Another study analyzed 57 patients with chemosensitive cancers, including 18 breast carcinomas, and showed that serum TC and LDL-C levels increased significantly after chemotherapy, with the exception of breast cancer patients. However, the TG level was only significantly increased in breast cancer patients after effective chemotherapy[23]. Furthermore, the changes in the levels of all these lipids and lipoproteins were reversible[23], which is somewhat consistent with the results from our study. Another study examined the metabolic changes in breast cancer patients who received chemotherapy and showed significant increases in TC, TG and LDL-C levels[24]. Overall, TG appears to be a sensitive measurement to determine the effect of adjuvant chemotherapy on women with breast cancer[25], and was shown to be an important and independent predictor of coronary heart disease, as a high TG level is a risk factor for cardiovascular complications in breast cancer patients[26, 27].

For HCY levels, several previous studies showed that serum homocysteine decreased after chemotherapy regimens including methotrexate[28, 29]. However, some studies presented constant HCY levels after chemotherapy in lung cancer patients and germ cell tumor patients[30, 31]. Thus, the change of HCY levels after chemotherapy is possibly associated with chemotherapeutic agents. This study showed a significant increase in UA levels in 6 months after chemotherapy, while the levels were constant during chemotherapy, indicating a delayed elevation of UA after chemotherapy. A study presented a similar result that UA levels had no significant change in the short-term after chemotherapy in breast cancer patients[32]. For the long-term after chemotherapy, the delayed increase in UA levels was possibly caused by the effect of chemotherapy on renal function. Because the lipid profiles change, chemotherapy agents affect the metabolism of lipid species by regulating the expression of genes involved in lipid metabolism in liver cells[33]. In addition, the improvements in health awareness, increased exercise, and changes in dietary habit are also potential explanations for these findings[24].

Regarding the use of different chemotherapy regimens by the adjuvant chemotherapy group, when we considered BMI as a confounding factor, the HDL-C level decreased to different extents in patients treated with different chemotherapy regimens, the combination of anthracycline with taxane exerted a greater effect on the HDL-C level. However, the effect size indicated that the different effects of these 3 different chemotherapy regimens on HDL-C levels were small. In previous studies, taxane-containing chemotherapy has been proven to induce dyslipidemia, which reduces the plasma HDL-C level and increases the plasma hydroperoxide level[18, 34]. In addition, cisplatin-based chemotherapy has also been confirmed to temporarily alter the plasma lipid levels[35]. The potential explanations for the different effects of several chemotherapy agents include the different mechanisms of chemotherapy agents. HDL-C levels were significantly reduced after chemotherapy in a previous study, and when the authors investigated the effects of individual chemotherapy agents on the expression of genes involved in lipoprotein metabolism in liver cells, doxorubicin downregulated the expression PPARγ (peroxisomal proliferator-activated receptor γ), liver X receptor α (LXRα), and ATP binding cassette transporter A1 (ABCA1). Meanwhile, cyclophosphamide or paclitaxel did not affect the ABCA1 level[33, 36].

Premenopausal and postmenopausal women have different statuses of lipid metabolism, and dyslipidemia is more common in postmenopausal women[14]. Based on the results of the analysis stratified by age, different age groups showed different changes in lipid profiles during chemotherapy. Chemotherapy appeared to exert a greater effect on younger breast cancer patients. Lipid metabolism is associated with sex hormones[37], and the changes in lipid levels after chemotherapy correlate with changes in menstruation[38]. Since younger patients have higher levels of sex hormones and a better lipid metabolism status, the plasma lipid levels are more sensitive to chemotherapy agents.

A limitation of this study is that it is a retrospective and single-center study, although we have equilibrated the baseline characteristics. We have also adjusted for confounding factors, such as age, baseline lipid levels and body mass index, and have excluded divergent data, but limitations still existed. Many factors influence the serum lipid levels, including diet and lifestyle, and the differences are difficult to control and balance. Furthermore, age is a probable factor contributing to the different sex hormone levels in patients. One factor that has been shown to affect lipid metabolism is the menstrual status and serum sex hormone levels, thus it could be better to stratify participants as pre-menopausal and post-menopausal. The use of other adjuvant therapies to treat breast cancer, such as tamoxifen treatment after chemotherapy, also affects the lipid levels [38]. Since we had discussed the potential explanations for changes in lipid profiles throughout chemotherapy, the specific explanations for these changes require further study and confirmation. Furthermore, since we only analyzed lipid levels 6 months after chemotherapy completion, the long-term effects of chemotherapy require further study. In addition, the small population remains a limitation of this study, and a subsequent multicenter randomized controlled trial should be performed to confirm the results and produce additional findings.

## Conclusions

Overall, TG, LDL-C and HDL-C levels changed to a worse status during chemotherapy, and the levels of LDL-C and HDL-C were restored after chemotherapy completion, while TG levels were still higher than the baseline level, showing an adverse temporary effect of chemotherapy. For patients treated with different chemotherapy regimens, only the HDL-C level differed among the different regimens, and the combination of anthracycline and taxane exerted a greater effect on decreasing the HDL-C level. Therefore, the different effects of different chemotherapy regimens were not distinct. Moreover, younger breast cancer patients were more sensitive to chemotherapy agents in this study.

## Supporting information

S1 Fig Diagram of included and excluded participants in this lipid analysis. (TIFF)

S1 File Minimal anonymized data set. (XLSX)

S2 File A manuscript showing changes using track changes. (DOCX)

S3 File Editorial certificate. (PDF)
