# Supplementary figures and images for: Changes in lipid profiles during and after (neo)adjuvant chemotherapy in women with early-stage breast cancer: A retrospective study
Source: PLoS One. 2019 Aug 29;14(8):e0221866. doi: 10.1371/journal.pone.0221866 (PMC6715243; doi:10.1371/journal.pone.0221866)

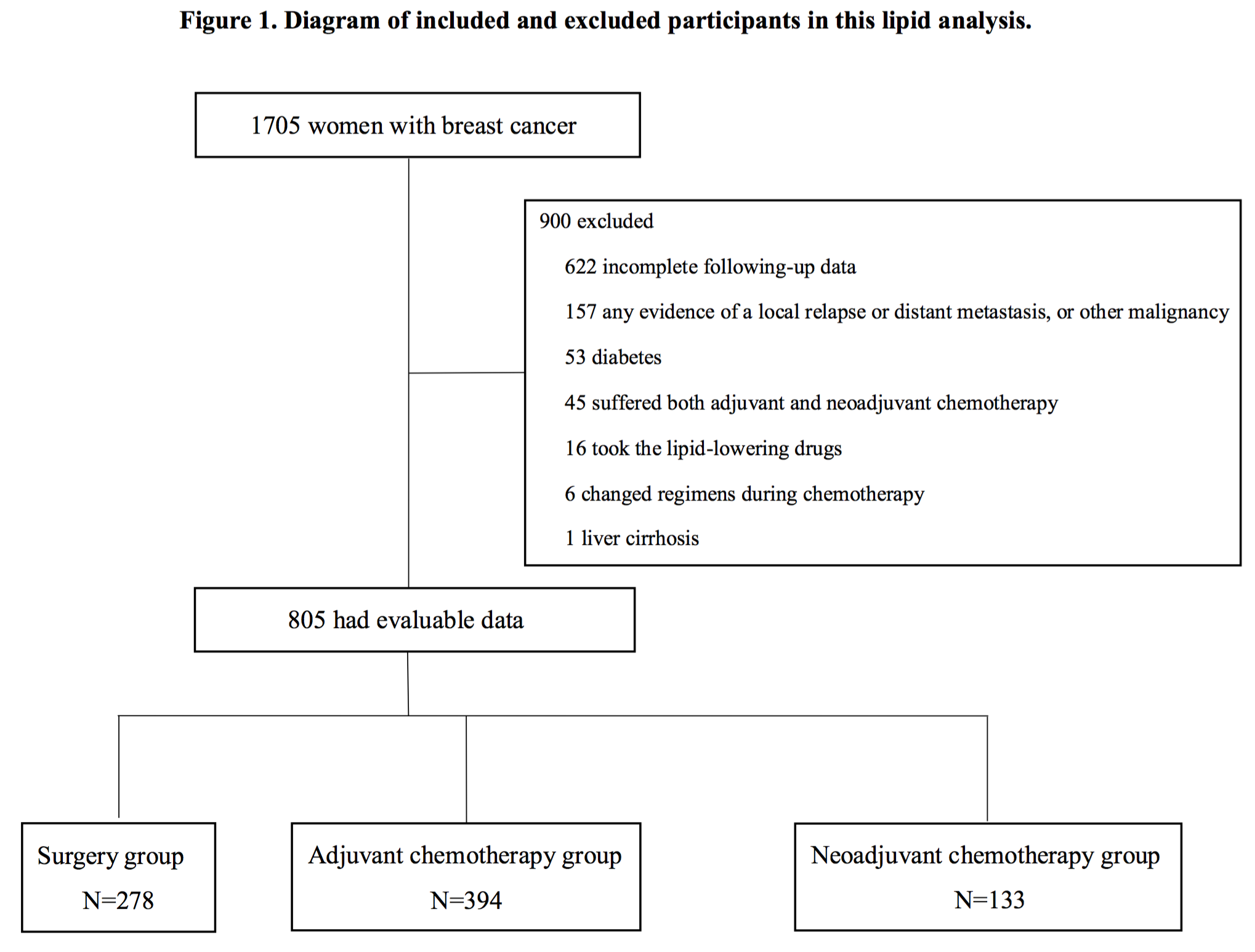

Supplement: S1 Fig — (TIFF) [file pone.0221866.s001.tiff]
