# Supplementary material for: Changes in lipid profiles during and after (neo)adjuvant chemotherapy in women with early-stage breast cancer: A retrospective study
Source: PLoS One. 2019 Aug 29;14(8):e0221866. doi: 10.1371/journal.pone.0221866 (PMC6715243; doi:10.1371/journal.pone.0221866)
Supplement: S2 File — (DOCX) [file pone.0221866.s003.docx]

**Changes in lipid profiles during and after (neo)adjuvant chemotherapy in women with early-stage breast cancer: a retrospective study**

Wei Tian^1,^ ^¶^, Yihan Yao^1, ¶^, Guocai Fan^2^, Yunxiang Zhou^1^, Miaowei Wu ^1^, Dong Xu^1^, Yongchuan Deng^1, *^

^1^ Department of Surgical Oncology, the Second Affiliated Hospital, School of Medicine, Zhejiang University, Hangzhou, Zhejiang, China

^2^ Department of Breast surgery, the people’s hospital of Suichang county, Lishui, Zhejiang, China

^¶^These authors contributed equally to this work.

^*^Corresponding author (E-mail: [dyc001@zju.edu.cn)](mailto:dyc001@zju.edu.cn))

**Abstract**

Many different treatments may affect the serum lipid profiles of breast cancer patients. This study analyzed serum lipid levels at different periods during treatment to observe the changes in lipid profiles during and after chemotherapy and to compare the different effects of different chemotherapy regimens on serum lipid profiles. A total of 805 patients were included in this study. We measured the lipid profiles of patients who received surgery without chemotherapy prior to the operation and at 3, 6 and 12 months after operation. In addition, in patients who underwent chemotherapy, the lipid profiles were measured prior to chemotherapy, prior to the last cycle of chemotherapy and 6 months after chemotherapy. Lipid profile measurements included total cholesterol (TC), triglycerides (TG), low-density lipoprotein cholesterol (LDL-C), high-density lipoprotein cholesterol (HDL-C), homocysteine (HCY), and uric acid (UA). (Neo)Adjuvant chemotherapy exerted a temporary effect on lipid levels (manifested as increased TG and LDL-C levels, and decreased HDL-C levels, particularly in the adjuvant chemotherapy group) during the chemotherapy periods. However, this influence was not sustained, as the lipid profiles levels were generally restored to baseline levels 6 months after chemotherapy completion. Different age groups showed different changes in lipid levels that were influenced by chemotherapy. The younger group (20-40 years old) showed a greater increase in TC and LDL-C levels during chemotherapy than the 41-65-year-old group. Chemotherapy exerts a temporary effect, and the effects of different regimens on lipid levels are similar. Furthermore, lipid profiles in younger women may be more sensitive to chemotherapy.

**Introduction**

Breast cancer is the most common malignant tumor and one of the main causes of cancer-related death in women[1-3]. Survival rates of breast cancer patients are increasing because of normative systemic treatment, including chemotherapy, targeted therapy, endocrine therapy, radiotherapy and various supportive treatments. Approximately 90% of breast cancer patients survive for at least 5 years after their initial diagnosis[4]. Some breast cancer patients eventually die of diseases other than breast cancer, especially cardiovascular disease (CVD)[5]. CVD and breast cancer share some predisposing risk factors, such as diet, obesity, and a sedentary lifestyle[6, 7]. On the other hand, long-term survivors may develop latent cardiac effects secondary to the cancer treatment (including chemotherapy, radiotherapy and targeted therapy). The above risk factors increase the mortality of CVD in older postmenopausal women (≥66 years old) who are long-term survivors (≥5 years). For these women, CVD exceeds breast cancer as the leading cause of death at 10 years after diagnosis[8]. Accordingly, we must focus on detecting the potential risk of CVD throughout process of diagnosis and management in these breast cancer patients.

For early-stage breast cancer patients, chemotherapy is an essential treatment to improve disease-free and overall survival. However, chemotherapy is associated with long-term side effects, including CVD. CVD (such as heart failure, myocardial ischemia, hypertension) has emerged as a common complication after chemotherapy[9, 10], not only due to the direct cardiotoxicity of chemotherapy drugs but also due to the impact on serum lipid levels. Anthracyclines induce irreversible cardiotoxicity, depending on its cumulative dose[11-13]. Regarding serum lipid levels, dyslipidemia (such as changes in high low-density lipoprotein cholesterol and low high-density lipoprotein cholesterol and hypertriglyceridemia) is a primary and major risk factor for atherosclerotic cardiovascular disease (ASCVD)[14, 15]. High serum cholesterol levels, especially high LDL-C, is causal and an independent risk factor for ASCVD[16, 17]. A low HDL-C level acts synergistically with other lipid risk factors to increase the ASCVD risk. Some studies have reported the effects of endocrine therapy and radiotherapy on serum lipid levels in breast cancer patients, including our group[18-21]. However, few studies have investigated the effect of chemotherapy on serum lipid levels. Therefore, a discussion of the effect of chemotherapy on serum lipid level during and after the completion of chemotherapy is important. Furthermore, researchers have not clearly determined whether chemotherapy treatments affect lipid profiles differently. This retrospective study analyzed the changes in lipid profiles during and after chemotherapy, and compared the different effects of different chemotherapy regimens on serum lipid profiles.

**Material and methods**

**Study participants and design**

Overall, we retrospectively analyzed breast cancer patients who underwent surgery, were treated with or without (neo)adjuvant therapy and attended follow-up visits at the Second Affiliated Hospital of Zhejiang University from January 1, 2012, to December 31, 2017. We included the patients on November 4, 2018, and the dates of data collection ranged from November 4, 2018 to November 15, 2018. Exclusion criteria included patients older than 65 years, patients who had dyslipidemia or took drugs affecting lipid levels, patients diagnosed with other malignant tumors or breast cancer recurrence or metastasis, patients with certain cardiovascular diseases (including coronary artery disease and stroke), diabetes mellitus or who took drugs affecting blood glucose levels.

All included patients accepted radical surgery. They were administered neoadjuvant or adjuvant chemotherapy according to clinicopathological manifestations and molecular subtypes. Hormone receptor (HR)-positive patients received adjuvant endocrine therapy, and HER-2-positive patients received trastuzumab concurrent with chemotherapy.

Patients were stratified into the following groups based on different treatments: a surgery group (received surgery but not chemotherapy), adjuvant chemotherapy group (received adjuvant chemotherapy after surgery) and neoadjuvant chemotherapy group (received neoadjuvant chemotherapy before surgery). For chemotherapy groups, participants were further divided into an anthracycline chemotherapy group (EC regimen), taxane chemotherapy group (TC, TCH or PH) and anthracycline plus taxane group (TEC or EC followed by P/T(H)). Medicine and disease histories, pathological results, immunohistochemistry results, tumor sizes, surgery approach and laboratory test results were obtained for each participant at baseline. The blood lipid levels were collected and analyzed from the database of the Second Affiliated Hospital of Zhejiang University. The levels of total cholesterol (TC), triglycerides (TG), low-density lipoprotein cholesterol (LDL-C), high-density lipoprotein cholesterol (HDL-C), homocysteine (HCY), and uric acid (UA), as well as the body mass index (BMI), were analyzed 4 times in the surgery group (prior to the operation and 3, 6 and 12 months after the operation) and 3 times in the chemotherapy groups (prior to chemotherapy, prior to the last cycle of chemotherapy and 6 months after chemotherapy).

Researchers had advised patients presenting substantial increases in blood lipid levels to seek a clinical consultation at the cardiovascular department during the follow-up period.

This study has been approved by the Ethics Committee of the Second Affiliated Hospital of Zhejiang University School of Medicine. In addition, the Ethics Committee waived the requirement for consent.

**Plasma lipid measurements**

LDL-C, HDL-C, HCY and UA levels were detected using direct methods, and the glycerol-phosphoric acid oxidase peroxidase method was used to measure the serum TG level. The cholesterol oxidase method was used to determine the serum TC level, as described previously[19].

**Statistical analyses**

Statistical analyses were performed using SPSS software version 20.0. Statistical descriptions, including means, standard deviations (SD), ranges and percentages, were used to characterize the eligible participants. Correlation test was used to investigate the correlations of different lipid measurements. A repeated measurement analysis of variance (RMANOVA) was performed to compare the differences in lipid levels at different time points before, during and after the interventions. The main effects included in the RMANOVA were the treatments patients received, including surgery, adjuvant chemotherapy and neoadjuvant chemotherapy. In addition, analysis of covariance was used to analyze the differences in changes in lipid levels between patients receiving different chemotherapy regimens and to analyze the differences in lipid levels between two year groups. As BMI levels also affected the lipid profiles, we also included BMI as a concomitant confounding variable in the analysis of covariance. Because multiple comparisons were performed, significance levels were adjusted in different groups.

**Results**

**Study population**

A total of 1705 patients were enrolled in this study, of which 805 had evaluable data and satisfied the inclusion criteria (Fig 1). Of the 805 participants, patients were divided into 3 groups based on the different treatments received: a surgery group (n=278), adjuvant chemotherapy group (n=394) and neoadjuvant chemotherapy group (n=133). The baseline characteristics of 3 groups of patients are shown in Table 1. Patients’ demographics and baseline characteristics were balanced among three groups.

**Fig 1. Diagram of included and excluded participants in this lipid analysis.**

**Table 1. Baseline characteristics of patients**

| Characteristic | Surgery (n=278) | Adjuvant chemotherapy (n=394) | Neoadjuvant chemotherapy (n=133) |
| --- | --- | --- | --- |
| Age, years  Mean (range) | 48.8 (23-65) | 48.9 (26-65) | 49.3 (26-65) |
| BMI, kg/m^2^  Mean (SD) | 22.55 (3.00) | 22.60 (2.78) | 23.27 (3.14) |
| Type of surgery |  |  |  |
| Breast-conserving | 103 (37.0%) | 152 (38.6%) | 25 (18.8%) |
| Mastectomy | 175 (63.0%) | 242 (61.4%) | 105 (78.9%) |
| Unknown | 0 | 0 | 3^*^ (2.3%) |
| Depth of tumor invasion (T) |  |  |  |
| Tis | 144 (51.8%) | 6 (1.5%) | 0 |
| T1 | 109 (39.2%) | 260 (66.0%) | 61 (45.9%) |
| T2 | 21 (7.6%) | 118 (30.0%) | 65 (48.9%) |
| T3-T4 | 1 (0.4%) | 4 (1.0%) | 7 (5.2%) |
| Unknown | 3^#^ (1.0%) | 6^#^ (1.5%) | 0 |
| Lymph node metastasis (N) |  |  |  |
| N0 | 267 (96.0%) | 273 (69.3%) | 21 (15.8%) |
| N1 | 11 (4.0%) | 100 (25.4%) | 94 (70.7%) |
| N2-N3 | 0 | 21 (5.3%) | 18 (13.5%) |
| ER status |  |  |  |
| Negative | 52 (18.7%) | 107 (27.2%) | 53 (39.8%) |
| Positive | 225 (80.9%) | 279 (70.8%) | 77 (57.9%) |
| Unknown | 1 (0.4%) | 8 (2.0%) | 3 (2.3%) |
| PR status |  |  |  |
| Negative | 51 (18.3%) | 135 (34.3%) | 69 (51.9%) |
| Positive | 226 (81.3%) | 252 (63.9%) | 61 (45.9%) |
| Unknown | 1 (0.4%) | 7 (1.8%) | 3 (2.2%) |
| Her-2 status |  |  |  |
| Negative | 189 (68.0%) | 258 (65.5%) | 85 (63.9%) |
| Positive | 61 (21.9%) | 134 (34.0%) | 47 (35.3%) |
| Unknown | 28^&^ (10.1%) | 2^#^ (0.5%) | 1^#^ (0.8%) |
| Ki-67 status |  |  |  |
| <20% | 167 (60.1%) | 112 (28.4%) | 58 (43.6%) |
| $\geq$20% | 94 (33.8%) | 260 (66.0%) | 64 (48.1%) |
| Unknown | 17 (6.1%) | 22 (5.6%) | 11 (8.3%) |
| Endocrine therapy |  |  |  |
| Yes | 228 (82.0%) | 285 (72.3%) | 82 (61.7%) |
| No | 50 (18.0%) | 109 (27.7%) | 51 (38.3%) |
| Radiation therapy |  |  |  |
| Yes | 101 (36.3%) | 213 (54.1%) | 107 (80.5%) |
| No | 177 (63.7%) | 181 (45.9%) | 26 (19.5%) |

BMI: Body mass index; SD: Standard deviation; ER: Estrogen receptor; PR: Progesterone receptor

^*^: Had not received the operation for breast cancer at the Second Affiliated Hospital of Zhejiang University after neoadjuvant chemotherapy.

^#^: No information was available.

^&^: No further FISH test in many patients with intraductal carcinoma.

**Lipid profiles of the surgery group**

The mean lipid levels, standard deviations and the comparisons between different time points in the surgery group are shown in Table 2. Clearly, the TG and UA levels increased significantly after surgery (3 and 6 months after the operation for TG levels and 3, 6, and 12 months after the operation for UA levels), while TC, LDL-C and HDL-C levels decreased significantly after surgery (3, 6, and 12 months after operation for TC levels, 3 and 12 months after the operation for LDL-C levels and 3 months after the operation for HDL-C levels). HCY and BMI levels were not significantly altered from 3-12 months after surgery.

**Table 2. Changes in the levels of 7 parameters in the surgery group**

| Surgery Group | | Preoperation | 3 months postoperation | 6 months postoperation | 12 months postoperation |
| --- | --- | --- | --- | --- | --- |
| TC | Mean±SD | 5.04±0.98 | 4.80±0.96^*^ | 4.85±0.99^*^ | 4.77±0.96^*^ |
| TG | Mean±SD | 1.43±1.13 | 1.77±1.27^*^ | 1.72±1.14^*^ | 1.65±1.11 |
| LDL-C | Mean±SD | 2.68±0.73 | 2.56±0.76^*^ | 2.57±0.78 | 2.50±0.77^*^ |
| HDL-C | Mean±SD | 1.40±0.34 | 1.33±0.34^*^ | 1.36±0.32 | 1.40±0.34 |
| HCY | Mean±SD | 10.42±6.51 | 10.04±5.64 | 9.82±4.79 | 10.34±4.14 |
| UA | Mean±SD | 262.41±55.58 | 285.73±65.60^*^ | 287.70±59.49^*^ | 298.11±59.62^*^ |
| BMI | Mean±SD | 22.57±2.96 | 22.58±2.89 | 22.59±2.91 | 22.52±2.90 |

TC: total cholesterol (mmol/L); TG: triglycerides (mmol/L); LDL-C: low-density lipoprotein cholesterol (mmol/L); HDL-C: high-density lipoprotein cholesterol (mmol/L); HCY: homocysteine (µmol/L); UA: uric acid (µmol/L); BMI: body mass index; SD: standard deviation

^*^ P<0.0167(0.05/3) compared with the baseline level (preoperation level)

**Lipid profiles of the adjuvant chemotherapy and neoadjuvant chemotherapy groups**

The mean lipid levels, standard deviations and the comparisons between different time points in the adjuvant chemotherapy and neoadjuvant chemotherapy groups are presented in Table 3 and Table 4, respectively. The result of correlation test showed that these lipid measurements were correlated to each other except HCY levels. For the adjuvant chemotherapy group, the TG and LDL-C levels and BMI increased significantly during chemotherapy, while HDL-C levels decreased significantly during chemotherapy. Furthermore, when the levels of these lipids were analyzed 6 months after chemotherapy, the TC and LDL-C levels decreased and were even lower than the prechemotherapy levels. In contrast, HDL-C and HCY levels increased after chemotherapy completion and were restored to baseline levels. In addition, TG levels and BMI were still higher than the prechemotherapy levels.

For the neoadjuvant chemotherapy group, the LDL-C level increased significantly during chemotherapy, showing a similar trend to the adjuvant chemotherapy group. However, the increase in TG and UA levels during chemotherapy was not significant, but at 6 months after chemotherapy completion, TG and UA levels were significantly higher than the baseline values.

**Table 3. Changes in the levels of 7 parameters in the adjuvant chemotherapy group**

| Adjuvant chemotherapy Group | | Prechemotherapy | Prior to the last cycle of chemotherapy | 6 m after chemotherapy |
| --- | --- | --- | --- | --- |
| TC | Mean±SD | 5.10±1.09 | 5.20±0.98 | 4.95±1.02^*#^ |
| TG | Mean±SD | 1.64±0.96 | 2.12±1.37^*^ | 1.97±1.69^*^ |
| LDL-C | Mean±SD | 2.86±0.76 | 2.97±0.78^*^ | 2.67±0.76^*#^ |
| HDL-C | Mean±SD | 1.34±0.32 | 1.21±0.31^*^ | 1.35±0.34^#^ |
| HCY | Mean±SD | 9.43±2.38 | 9.00±2.44 | 9.58±2.48^#^ |
| UA | Mean±SD | 270.05±55.12 | 272.80±61.22 | 291.51±58.70^*#^ |
| BMI | Mean±SD | 22.44±2.71 | 22.82±2.86^*^ | 22.69±2.82^*^ |

TC: total cholesterol (mmol/L); TG: triglycerides (mmol/L); LDL-C: low-density lipoprotein cholesterol (mmol/L); HDL-C: high-density lipoprotein cholesterol (mmol/L); HCY: homocysteine (µmol/L); UA: uric acid (µmol/L); BMI: body mass index; 6 m: 6 months; SD: standard deviation

^*^ P<0.025(0.05/2) compared with the baseline level (prechemotherapy level)

^#^ P<0.025(0.05/2) compared with the level measured prior to the last cycle of chemotherapy

**Table 4. Changes in the levels of 7 parameters in the neoadjuvant chemotherapy group**

| Neoadjuvant chemotherapy group | | Prechemotherapy | Prior to the last cycle of chemotherapy | 6 m after chemotherapy |
| --- | --- | --- | --- | --- |
| TC | Mean±SD | 5.14±1.08 | 5.34±1.13 | 5.19±1.20 |
| TG | Mean±SD | 1.42±0.78 | 1.97±1.02 | 1.86±0.83^*^ |
| LDL-C | Mean±SD | 2.82±0.78 | 3.14±0.82^*^ | 2.92±0.88 |
| HDL-C | Mean±SD | 1.33±0.31 | 1.17±0.24 | 1.33±0.28 |
| HCY | Mean±SD | 11.44±9.46 | 9.65±2.57 | 10.56±2.45 |
| UA | Mean±SD | 270.78±59.44 | 274.28±62.22 | 301.06±60.66^*^ |
| BMI | Mean±SD | 23.07±3.04 | 23.16±2.87 | 23.22±2.88 |

TC: total cholesterol (mmol/L); TG: triglycerides (mmol/L); LDL-C: low-density lipoprotein cholesterol (mmol/L); HDL-C: high-density lipoprotein cholesterol (mmol/L); HCY: homocysteine (µmol/L); UA: uric acid (µmol/L); BMI: body mass index; 6 m: 6 months; SD: standard deviation

^*^ P<0.025(0.05/2) compared with the baseline level (prechemotherapy level)

These data show the lipid profiles of the neoadjuvant and adjuvant chemotherapy groups. We subsequently analyzed the lipid profiles in groups stratified according to the use of different chemotherapeutic regimens. However, because of the limited number of patients in the anthracycline chemotherapy regimen subgroup of the neoadjuvant chemotherapy group (n=2), the participants in the neoadjuvant chemotherapy group were not further analyzed according to the use of different chemotherapeutic regimens.

**Lipid profiles of patients receiving different chemotherapy regimens in the adjuvant chemotherapy group**

Comparisons of lipid profiles between the 3 different adjuvant chemotherapy regimen groups are presented in Table 5.

**Table 5. Levels of 7 parameters in patients receiving different chemotherapy regimens throughout chemotherapy**

| Parameter | Regimen | Prechemotherapy | Prior to the last cycle of chemotherapy | 6 m after chemotherapy |
| --- | --- | --- | --- | --- |
|  |  | Mean$\pm$SD | Mean$\pm$SD | Mean$\pm$SD |
| TC | Anthracycline | 5.09$\pm$1.09 | 5.25$\pm$1.15 | 4.93$\pm$0.80 |
|  | Taxane | 5.10$\pm$1.09 | 5.18$\pm$0.89 | 4.87$\pm$1.07^*^ |
|  | Anthracycline+taxane | 5.10$\pm$1.09 | 5.19$\pm$1.02 | 5.05$\pm$1.05 |
| TG | Anthracycline | 1.68$\pm$0.81 | 2.14$\pm$1.52^*^ | 2.13$\pm$2.05 |
|  | Taxane | 1.60$\pm$1.00 | 2.04$\pm$1.13^*^ | 2.00$\pm$1.91^*^ |
|  | Anthracycline+taxane | 1.68$\pm$0.98 | 2.21$\pm$1.56^*^ | 1.85$\pm$1.12^*^ |
| LDL-C | Anthracycline | 2.89$\pm$0.79 | 3.05$\pm$0.91 | 2.62$\pm$0.64^*^ |
|  | Taxane | 2.84$\pm$0.74 | 2.92$\pm$0.69 | 2.59$\pm$0.76^*&^ |
|  | Anthracycline+taxane | 2.87$\pm$0.78 | 3.01$\pm$0.83^*^ | 2.78$\pm$0.80 |
| HDL-C | Anthracycline | 1.39$\pm$0.41 | 1.26$\pm$0.29^*&^ | 1.38$\pm$0.36 |
|  | Taxane | 1.35$\pm$0.32 | 1.24$\pm$0.34^*&^ | 1.34$\pm$0.35 |
|  | Anthracycline+taxane | 1.32$\pm$0.27 | 1.16$\pm$0.27^*^ | 1.34$\pm$0.30 |
| HCY | Anthracycline | 9.11$\pm$2.38 | 9.39$\pm$2.86 | 8.81$\pm$2.05 |
|  | Taxane | 9.60$\pm$2.35 | 9.10$\pm$2.23^*^ | 9.84$\pm$2.13^#^ |
|  | Anthracycline+taxane | 9.36$\pm$2.42 | 8.72$\pm$2.51^*^ | 9.59$\pm$2.96 |
| UA | Anthracycline | 283.41$\pm$60.69 | 296.22$\pm$77.25^*^ | 293.67$\pm$54.52 |
|  | Taxane | 266.48$\pm$51.59 | 268.12$\pm$58.50^#^ | 292.36$\pm$57.36^*^ |
|  | Anthracycline+taxane | 268.77$\pm$56.57 | 268.52$\pm$54.62^#^ | 289.47$\pm$62.50^*^ |
| BMI | Anthracycline | 22.27$\pm$2.55 | 22.37$\pm$2.67 | 22.43$\pm$2.74 |
|  | Taxane | 22.46$\pm$2.86 | 22.92$\pm$2.92^*＃^ | 22.66$\pm$2.89 |
|  | Anthracycline+taxane | 22.50$\pm$2.60 | 22.89$\pm$2.88^*^ | 22.85$\pm$2.77^*^ |

TC: total cholesterol (mmol/L); TG: triglycerides (mmol/L); LDL-C: low-density lipoprotein cholesterol (mmol/L); HDL-C: high-density lipoprotein cholesterol (mmol/L); HCY: homocysteine (µmol/L); UA: uric acid (µmol/L); BMI: body mass index; 6 m: 6 months; SD: standard deviation

^*^: P<0.05 compared with baseline levels (prechemotherapy levels)

^#^: P<0.05 compared with the group receiving the anthracycline chemotherapy regimen

^&^: P<0.05 compared with the group receiving the anthracycline + taxane chemotherapy regimen

TC levels were slightly increased before the last cycles of chemotherapy compared with the prechemotherapy levels in all three regimen subgroups. At 6 months postchemotherapy, TC levels returned to baseline levels, except in the taxane group, in which the levels were significantly decreased compared with baseline levels (P<0.05). The three different chemotherapy regimens showed no significant difference in the change in TC levels.

TG levels increased significantly during chemotherapy in all 3 chemotherapy regimen groups. At 6 months after chemotherapy completion, the TG levels were slightly decreased but were still higher than baseline levels (prechemotherapy level) in all three groups, particularly in the groups receiving the taxane and anthracycline combined with taxane chemotherapy regimens (P<0.05). Furthermore, a significant difference in changes in TG levels was not observed between the groups receiving the three different chemotherapy regimens during chemotherapy and after chemotherapy completion.

The LDL-C levels were significantly increased during chemotherapy in the anthracycline plus taxane group (P<0.05), but were restored to baseline levels 6 months after chemotherapy completion. In the anthracycline group and taxane group, the LDL-C levels were slightly increased (but not significantly) during chemotherapy and decreased thereafter. Significantly lower LDL-C levels were observed 6 months after chemotherapy completion than at baseline in these two groups (P<0.05). Similarly, a significant difference in the change in LDL-C levels was not observed between patients receiving the three chemotherapy regimens during chemotherapy; however, at 6 months after chemotherapy completion, the group treated with the combination of anthracycline and taxane presented a higher LDL-C level than the taxane group (P<0.05).

The HDL-C levels decreased significantly during chemotherapy in all 3 regimen groups, and a more substantial decrease was observed in the group treated with the combination of anthracycline and taxane compared with the other two groups. At 6 months after chemotherapy completion, the levels were nearly restored to baseline levels.

The HCY levels decreased significantly in the taxane group and anthracycline plus taxane group during chemotherapy (P<0.05) and were restored to baseline levels 6 months after chemotherapy completion. However, a significant decrease was not observed in the anthracycline group. In addition, a significant difference in HCY levels was not observed between the different chemotherapy regimens. Furthermore, the effect size (partial eta squared) of chemotherapy regimens on HDL-C levels was 0.02.

The UA level was only significantly increased in the anthracycline chemotherapy regimen group during chemotherapy. During chemotherapy, the UA level in anthracycline group was significantly higher than the taxane group and anthracycline plus taxane group. Furthermore, at 6 months after chemotherapy completion, the UA level in the anthracycline group was restored to the baseline level, while the levels in the other 2 groups were higher than baseline levels.

The BMI increased significantly in the taxane group and anthracycline plus taxane group during chemotherapy, while the BMI of the anthracycline group was not significantly increased throughout and after chemotherapy. In addition, the BMI of the taxane group was restored to the baseline level 6 months after chemotherapy completion, while the BMI of the anthracycline plus taxane group did not change at this time point.

**Lipid profiles of the adjuvant chemotherapy group after stratification into different age groups**

Considering the differences in lipid metabolism in women of different ages, the adjuvant chemotherapy group was further stratified into a 20-40-year-old group and a 41-65-year-old group. The mean lipid levels, standard deviations and comparisons at different time points are presented in Table 6.

**Table 6. Levels of 7 parameters in different age groups throughout chemotherapy**

| Parameter | Group | Prechemotherapy | Prior to the last cycle of chemotherapy | | 6 m after chemotherapy | |
| --- | --- | --- | --- | --- | --- | --- |
|  |  | Mean$\pm$SD | Mean$\pm$SD | Change | Mean$\pm$SD | Change |
| TC | 20-40 | 4.63$\pm$1.01 | 5.01$\pm$0.96 | 0.39 | 4.48$\pm$0.79 | -0.03 |
|  | 41-65 | 5.26$\pm$1.11^*^ | 5.25$\pm$0.96 | -0.01^#^ | 5.04$\pm$1.04 | -0.18 |
| TG | 20-40 | 1.26$\pm$0.63 | 1.93$\pm$1.48 | 0.67 | 1.68$\pm$1.92 | 0.47 |
|  | 41-65 | 1.75$\pm$0.98^*^ | 2.14$\pm$1.25 | 0.39 | 2.02$\pm$1.63 | 0.30 |
| LDL-C | 20-40 | 2.50$\pm$0.77 | 2.79$\pm$0.77 | 0.29 | 2.24$\pm$0.63 | -0.19 |
|  | 41-65 | 2.97$\pm$0.77^*^ | 3.03$\pm$0.76 | 0.06^#^ | 2.75$\pm$0.76 | -0.20 |
| HDL-C | 20-40 | 1.35$\pm$0.28 | 1.27$\pm$0.36 | -0.08 | 1.41$\pm$0.38 | 0.08 |
|  | 41-65 | 1.35$\pm$0.33 | 1.20$\pm$0.29 | -0.15 | 1.33$\pm$0.33 | -0.01 |
| HCY | 20-40 | 8.54$\pm$2.26 | 8.02$\pm$1.56 | -0.55 | 8.49$\pm$1.66 | -0.23 |
|  | 41-65 | 9.69$\pm$2.32^*^ | 9.43$\pm$3.02 | -0.37 | 9.79$\pm$2.56 | 0.22 |
| UA | 20-40 | 260.97$\pm$55.73 | 264.31$\pm$54.21 | 3.34 | 278.72$\pm$40.85 | 20.96 |
|  | 41-65 | 273.85$\pm$55.97 | 273.98$\pm$60.85 | 0.12 | 294.02$\pm$61.36 | 21.55 |
| BMI | 20-40 | 21.36$\pm$2.78 | 21.87$\pm$3.02 | 0.50 | 21.69$\pm$2.78 | 0.45 |
|  | 41-65 | 22.70$\pm$2.67^*^ | 23.04$\pm$2.80 | 0.34 | 22.89$\pm$2.79 | 0.21 |

Change indicates the absolute change from baseline levels. TC: total cholesterol (mmol/L); TG: triglycerides (mmol/L); LDL-C: low-density lipoprotein cholesterol (mmol/L); HDL-C: high-density lipoprotein cholesterol (mmol/L); HCY: homocysteine (µmol/L); UA: uric acid (µmol/L); BMI: body mass index; 6 m: 6 months; SD: standard deviation

^*^: P<0.05 compared with the level of the same parameter in the 20-40-year-old group at a specific time point

^#^: P<0.05 compared with the change in the level of the same parameter in the 20-40-year-old group at a specific time point

When we compared baseline (prechemotherapy) levels between the different age groups , the baseline TC, TG, LDL-C, and HCY levels and BMI of the 41-65-year-old group were higher than the 20-40-year-old group (P<0.05), while HDL-C and UA levels were not significantly different prior to chemotherapy.

In a comparison of the extent of the changes in the levels of the 7 parameters in different age groups during chemotherapy and after chemotherapy completion, TC and LDL-C levels showed a greater increase in the 20-40-year-old group during chemotherapy (P<0.05). Furthermore, the levels of both levels were restored to baseline levels at 6 months after chemotherapy completion.

**Discussion**

Based on the results from the present study, almost all serum lipid levels exhibited significant changes after surgery in the surgery group, with stable HCY levels and BMI. Surgery altered the serum lipid profiles. The changes in nutritional status, dietary structure and physical exercise, and the removal of a large quantity of adipose tissue during surgery may explain the changes in the lipid profiles of breast cancer patients after surgery. However, the detailed explanations for this phenomenon remain unclear and require further study.

In the adjuvant chemotherapy group, significant changes in the levels of some components of the lipid profile were observed during chemotherapy (TG and LDL-C levels increased, while HDL-C levels decreased), the changes in TC, HCY and UA levels after chemotherapy were not significant. However, at 6 months after chemotherapy completion, the HDL-C level was restored to the baseline level, the LDL-C level decreased to an even lower value than before chemotherapy, while the TG level was still significantly higher than the baseline level. changes observed during chemotherapy are partially consistent with some previous studies. Li, Xin and coworkers conducted a retrospective study to analyze the levels of lipids and lipoproteins before and after chemotherapy, and observed significantly higher TC, TG, and LDL-C levels in patients after chemotherapy than before chemotherapy, while the HDL-C level showed the opposite trend[22]. Another study analyzed 57 patients with chemosensitive cancers, including 18 breast carcinomas, and showed that serum TC and LDL-C levels increased significantly after chemotherapy, with the exception of breast cancer patients. However, the TG level was only significantly increased in breast cancer patients after effective chemotherapy[23]. Furthermore, the changes in the levels of all these lipids and lipoproteins were reversible[23], which is somewhat consistent with the results from our study. Another study examined the metabolic changes in breast cancer patients who received chemotherapy and showed significant increases in TC, TG and LDL-C levels[24]. Overall, TG appears to be a sensitive measurement to determine the effect of adjuvant chemotherapy on women with breast cancer[25], and was shown to be an important and independent predictor of coronary heart disease, as a high TG level is a risk factor for cardiovascular complications in breast cancer patients[26, 27]. Because the lipid profiles change, chemotherapy agents affect the metabolism of lipid species by regulating the expression of genes involved in lipid metabolism in liver cells[28]. In addition, the improvements in health awareness, increased exercise, and changes in dietary habit are also potential explanations for these findings[24].

Regarding the use of different chemotherapy regimens by the adjuvant chemotherapy group, when we considered BMI as a confounding factor, the HDL-C level decreased to different extents in patients treated with different chemotherapy regimens, the combination of anthracycline with taxane exerted a greater effect on the HDL-C level. In previous studies, taxane-containing chemotherapy has been proven to induce dyslipidemia, which reduces the plasma HDL-C level and increases the plasma hydroperoxide level[18, 29]. In addition, cisplatin-based chemotherapy has also been confirmed to temporarily alter the plasma lipid levels[30]. The potential explanations for the different effects of several chemotherapy agents include the different mechanisms of chemotherapy agents. HDL-C levels were significantly reduced after chemotherapy in a previous study, and when the authors investigated the effects of individual chemotherapy agents on the expression of genes involved in lipoprotein metabolism in liver cells, doxorubicin downregulated the expression PPARγ (peroxisomal proliferator-activated receptor γ), liver X receptor α (LXRα), and ATP binding cassette transporter A1 (ABCA1). Meanwhile, cyclophosphamide or paclitaxel did not affect the ABCA1 level[28, 31].

Premenopausal and postmenopausal women have different statuses of lipid metabolism, and dyslipidemia is more common in postmenopausal women[14]. Based on the results of the analysis stratified by age, different age groups showed different changes in lipid profiles during chemotherapy. Chemotherapy appeared to exert a greater effect on younger breast cancer patients. Lipid metabolism is associated with sex hormones[32], and the changes in lipid levels after chemotherapy correlate with changes in menstruation[33]. Since younger patients have higher levels of sex hormones and a better lipid metabolism status, the plasma lipid levels are more sensitive to chemotherapy agents.

A limitation of this study is that it is a retrospective and single-center study, although we have equilibrated the baseline characteristics. We have also adjusted for confounding factors, such as age, baseline lipid levels and body mass index, and have excluded divergent data, but limitations still existed. Many factors influence the serum lipid levels, including diet and lifestyle, and the differences are difficult to control and balance. Furthermore, age is a probable factor contributing to the different sex hormone levels in patients. One factor that has been shown to affect lipid metabolism is the menstrual status and serum sex hormone levels. The use of other adjuvant therapies to treat breast cancer, such as tamoxifen treatment after chemotherapy, also affects the lipid levels [33]. Since we had discussed the potential explanations for changes in lipid profiles throughout chemotherapy, the specific explanations for these changes require further study and confirmation. Furthermore, since we only analyzed lipid levels 6 months after chemotherapy completion, the long-term effects of chemotherapy require further study. In addition, the small population remains a limitation of this study, and a subsequent multicenter randomized controlled trial should be performed to confirm the results and produce additional findings.

**Conclusions**

Overall, TG, LDL-C and HDL-C levels changed to a worse status during chemotherapy, and the levels of LDL-C and HDL-C were restored after chemotherapy completion, while TG levels were still higher than the baseline level, showing a temporary effect of chemotherapy. For patients treated with different chemotherapy regimens, only the HDL-C level differed among the different regimens, and the combination of anthracycline and taxane exerted a greater effect on decreasing the HDL-C level. Therefore, the different effects of different chemotherapy regimens were not distinct. Moreover, younger breast cancer patients were more sensitive to chemotherapy agents in this study.

**Acknowledgements**

The authors wish to thank all patients who participanted in this study, and we thank to the anonymous reviewers who help to improve the paper.

**Funding**

Wei Tian was supported by grants from the National Natural Science Foundation of China (grant nos. 81502598). Dong Xu was supported by grants from the National Natural Science Foundation of China (grant nos. 81773181).

**Availability of Data and Matarials**

The datasets used and analyzed during the current study are available from the corresponding author on reasonable request.

**Authors’ Contributions**

Wei Tian designed the research and writed the manuscript. Yihan Yao collected and analyzed the data, and writed the manuscript. Guocai Fan collected the data and analyzed the data. Yunxiang Zhou and Miaowei Wu collected the data. Dong Xu and Yongchuan Deng reviewed and edited the manuscript. Wei Tian and Yihan Yao contributed equally to the work. All authors read and approved the final manuscript.

**Ethics Approval and Consent to Participant**

This study was conducted in accordance with the guidelines of the 1975 Declaration of Helsinki. The need for informed patient consent was waived because of the retrospective nature of the study. The study protocol has been approved by the ethics committee of the Second Affiliated Hospital of Zhejiang University (no. 2018-130).

**Patient Consent for Publication**

All identifying information is removed in this manuscript.

**Competing Interests**

The authors declare that they have no competing interests.

**References:**

1. Chen W, Zheng R, Zhang S, Zeng H, Zuo T, Xia C, et al. Cancer incidence and mortality in China in 2013: an analysis based on urbanization level. Chinese journal of cancer research = Chung-kuo yen cheng yen chiu. 2017;29(1):1-10. Epub 2017/04/05. doi: 10.21147/j.issn.1000-9604.2017.01.01. PubMed PMID: 28373748; PubMed Central PMCID: PMCPMC5348470.

2. DeSantis CE, Ma J, Goding Sauer A, Newman LA, Jemal A. Breast cancer statistics, 2017, racial disparity in mortality by state. CA: a cancer journal for clinicians. 2017;67(6):439-48. Epub 2017/10/04. doi: 10.3322/caac.21412. PubMed PMID: 28972651.

3. Siegel RL, Miller KD, Jemal A. Cancer Statistics, 2017. CA: a cancer journal for clinicians. 2017;67(1):7-30. Epub 2017/01/06. doi: 10.3322/caac.21387. PubMed PMID: 28055103.

4. Cancer stat facts: female breast cancer. Surveillance, Epidemiology, and End Results Program website.

5. Bardia A, Arieas ET, Zhang Z, Defilippis A, Tarpinian K, Jeter S, et al. Comparison of breast cancer recurrence risk and cardiovascular disease incidence risk among postmenopausal women with breast cancer. Breast cancer research and treatment. 2012;131(3):907-14. Epub 2011/11/02. doi: 10.1007/s10549-011-1843-1. PubMed PMID: 22042368; PubMed Central PMCID: PMCPMC3582017.

6. Guenancia C, Lefebvre A, Cardinale D, Yu AF, Ladoire S, Ghiringhelli F, et al. Obesity As a Risk Factor for Anthracyclines and Trastuzumab Cardiotoxicity in Breast Cancer: A Systematic Review and Meta-Analysis. Journal of clinical oncology : official journal of the American Society of Clinical Oncology. 2016;34(26):3157-65. Epub 2016/07/28. doi: 10.1200/jco.2016.67.4846. PubMed PMID: 27458291; PubMed Central PMCID: PMCPMC5569689.

7. Wang YC, Graubard BI, Rosenberg MA, Kuntz KM, Zauber AG, Kahle L, et al. Derivation of background mortality by smoking and obesity in cancer simulation models. Medical decision making : an international journal of the Society for Medical Decision Making. 2013;33(2):176-97. Epub 2012/11/08. doi: 10.1177/0272989x12458725. PubMed PMID: 23132901; PubMed Central PMCID: PMCPMC3663442.

8. Abdel-Qadir H, Austin PC, Lee DS, Amir E, Tu JV, Thavendiranathan P, et al. A Population-Based Study of Cardiovascular Mortality Following Early-Stage Breast Cancer. JAMA cardiology. 2017;2(1):88-93. Epub 2016/10/13. doi: 10.1001/jamacardio.2016.3841. PubMed PMID: 27732702.

9. Matyszewski A, Czarnecka AM, Stachowiak P, Nowakowska M, Kornacewicz-Jach Z, Kasprzak JD, et al. Cardiac safety of systemic therapy in breast cancer patients with high risk of atherosclerosis complications. Future oncology (London, England). 2017;13(7):593-602. Epub 2016/10/28. doi: 10.2217/fon-2016-0425. PubMed PMID: 27784174.

10. Truong J, Yan AT, Cramarossa G, Chan KK. Chemotherapy-induced cardiotoxicity: detection, prevention, and management. The Canadian journal of cardiology. 2014;30(8):869-78. Epub 2014/07/30. doi: 10.1016/j.cjca.2014.04.029. PubMed PMID: 25064580.

11. Doyle JJ, Neugut AI, Jacobson JS, Grann VR, Hershman DL. Chemotherapy and cardiotoxicity in older breast cancer patients: a population-based study. Journal of clinical oncology : official journal of the American Society of Clinical Oncology. 2005;23(34):8597-605. Epub 2005/11/30. doi: 10.1200/jco.2005.02.5841. PubMed PMID: 16314622.

12. Ades F, Zardavas D, Pinto AC, Criscitiello C, Aftimos P, de Azambuja E. Cardiotoxicity of systemic agents used in breast cancer. Breast (Edinburgh, Scotland). 2014;23(4):317-28. Epub 2014/05/06. doi: 10.1016/j.breast.2014.04.002. PubMed PMID: 24794210.

13. Smith LA, Cornelius VR, Plummer CJ, Levitt G, Verrill M, Canney P, et al. Cardiotoxicity of anthracycline agents for the treatment of cancer: systematic review and meta-analysis of randomised controlled trials. BMC cancer. 2010;10:337. Epub 2010/07/01. doi: 10.1186/1471-2407-10-337. PubMed PMID: 20587042; PubMed Central PMCID: PMCPMC2907344.

14. Rodrigues Dos Santos C, Fonseca I, Dias S, Mendes de Almeida JC. Plasma level of LDL-cholesterol at diagnosis is a predictor factor of breast tumor progression. BMC cancer. 2014;14:132. Epub 2014/02/28. doi: 10.1186/1471-2407-14-132. PubMed PMID: 24571647; PubMed Central PMCID: PMCPMC3942620.

15. Smith DG. Epidemiology of dyslipidemia and economic burden on the healthcare system. The American journal of managed care. 2007;13 Suppl 3:S68-71. Epub 2007/07/11. PubMed PMID: 17596114.

16. Jellinger PS, Handelsman Y, Rosenblit PD, Bloomgarden ZT, Fonseca VA, Garber AJ, et al. AMERICAN ASSOCIATION OF CLINICAL ENDOCRINOLOGISTS AND AMERICAN COLLEGE OF ENDOCRINOLOGY GUIDELINES FOR MANAGEMENT OF DYSLIPIDEMIA AND PREVENTION OF CARDIOVASCULAR DISEASE. Endocrine practice : official journal of the American College of Endocrinology and the American Association of Clinical Endocrinologists. 2017;23(Suppl 2):1-87. Epub 2017/04/25. doi: 10.4158/ep171764.appgl. PubMed PMID: 28437620.

17. Huxley R, Lewington S, Clarke R. Cholesterol, coronary heart disease and stroke: a review of published evidence from observational studies and randomized controlled trials. Seminars in vascular medicine. 2002;2(3):315-23. Epub 2005/10/14. doi: 10.1055/s-2002-35402. PubMed PMID: 16222621.

18. Yeo W, Mo FKF, Pang E, Suen JJS, Koh J, Loong HHF, et al. Profiles of lipids, blood pressure and weight changes among premenopausal Chinese breast cancer patients after adjuvant chemotherapy. BMC women's health. 2017;17(1):55. Epub 2017/07/29. doi: 10.1186/s12905-017-0409-8. PubMed PMID: 28750616; PubMed Central PMCID: PMCPMC5532788.

19. Tian W, Wu M, Deng Y. Comparison of Changes in the Lipid Profiles of Eastern Chinese Postmenopausal Women With Early-Stage Breast Cancer Treated With Different Aromatase Inhibitors: A Retrospective Study. Clinical pharmacology in drug development. 2017. Epub 2017/12/30. doi: 10.1002/cpdd.420. PubMed PMID: 29287126.

20. Lu HH, Li Q, Xu BH, Zhang P, Yuan P, Wang JY, et al. [Effects of anastrozole on lipid metabolism in Chinese postmenopausal women with breast cancer]. Zhonghua zhong liu za zhi [Chinese journal of oncology]. 2011;33(7):520-5. Epub 2011/11/19. PubMed PMID: 22093630.

21. Shaikh S, Channa NA, Talpur FN, Younis M, Tabassum N. Radiotherapy improves serum fatty acids and lipid profile in breast cancer. Lipids in health and disease. 2017;16(1):92. Epub 2017/05/20. doi: 10.1186/s12944-017-0481-y. PubMed PMID: 28521812; PubMed Central PMCID: PMCPMC5437547.

22. Li X, Liu ZL, Wu YT, Wu H, Dai W, Arshad B, et al. Status of lipid and lipoprotein in female breast cancer patients at initial diagnosis and during chemotherapy. Lipids in health and disease. 2018;17(1):91. Epub 2018/04/22. doi: 10.1186/s12944-018-0745-1. PubMed PMID: 29678178; PubMed Central PMCID: PMCPMC5910614.

23. Alexopoulos CG, Pournaras S, Vaslamatzis M, Avgerinos A, Raptis S. Changes in serum lipids and lipoproteins in cancer patients during chemotherapy. Cancer chemotherapy and pharmacology. 1992;30(5):412-6. Epub 1992/01/01. PubMed PMID: 1505080.

24. Arpino G, De Angelis C, Buono G, Colao A, Giuliano M, Malgieri S, et al. Metabolic and anthropometric changes in early breast cancer patients receiving adjuvant therapy. Breast cancer research and treatment. 2015;154(1):127-32. Epub 2015/10/02. doi: 10.1007/s10549-015-3586-x. PubMed PMID: 26424165; PubMed Central PMCID: PMCPMC4621696.

25. Rzymowska J. Effect of cytotoxic chemotherapy on serum lipid levels in breast cancer patients. Pathobiology : journal of immunopathology, molecular and cellular biology. 1999;67(3):129-32. Epub 1999/07/08. doi: 10.1159/000028062. PubMed PMID: 10394133.

26. Patel A, Barzi F, Jamrozik K, Lam TH, Ueshima H, Whitlock G, et al. Serum triglycerides as a risk factor for cardiovascular diseases in the Asia-Pacific region. Circulation. 2004;110(17):2678-86. Epub 2004/10/20. doi: 10.1161/01.cir.0000145615.33955.83. PubMed PMID: 15492305.

27. Shenoy C, Klem I, Crowley AL, Patel MR, Winchester MA, Owusu C, et al. Cardiovascular complications of breast cancer therapy in older adults. The oncologist. 2011;16(8):1138-43. Epub 2011/07/09. doi: 10.1634/theoncologist.2010-0348. PubMed PMID: 21737575; PubMed Central PMCID: PMCPMC3228150.

28. Sharma M, Tuaine J, McLaren B, Waters DL, Black K, Jones LM, et al. Chemotherapy Agents Alter Plasma Lipids in Breast Cancer Patients and Show Differential Effects on Lipid Metabolism Genes in Liver Cells. PloS one. 2016;11(1):e0148049. Epub 2016/01/26. doi: 10.1371/journal.pone.0148049. PubMed PMID: 26807857; PubMed Central PMCID: PMCPMC4726544.

29. Panis C, Binato R, Correa S, Victorino VJ, Dias-Alves V, Herrera A, et al. Short infusion of paclitaxel imbalances plasmatic lipid metabolism and correlates with cardiac markers of acute damage in patients with breast cancer. Cancer chemotherapy and pharmacology. 2017;80(3):469-78. Epub 2017/07/10. doi: 10.1007/s00280-017-3384-8. PubMed PMID: 28689298.

30. Willemse PM, van der Meer RW, Burggraaf J, van Elderen SG, de Kam ML, de Roos A, et al. Abdominal visceral and subcutaneous fat increase, insulin resistance and hyperlipidemia in testicular cancer patients treated with cisplatin-based chemotherapy. Acta oncologica (Stockholm, Sweden). 2014;53(3):351-60. Epub 2013/08/21. doi: 10.3109/0284186x.2013.819116. PubMed PMID: 23957624.

31. Basso F, Freeman L, Knapper CL, Remaley A, Stonik J, Neufeld EB, et al. Role of the hepatic ABCA1 transporter in modulating intrahepatic cholesterol and plasma HDL cholesterol concentrations. Journal of lipid research. 2003;44(2):296-302. Epub 2003/02/11. doi: 10.1194/jlr.M200414-JLR200. PubMed PMID: 12576511.

32. Delgobo M, Agnes JP, Goncalves RM, Dos Santos VW, Parisotto EB, Zamoner A, et al. N-acetylcysteine and alpha-lipoic acid improve antioxidant defenses and decrease oxidative stress, inflammation and serum lipid levels in ovariectomized rats via estrogen-independent mechanisms. The Journal of nutritional biochemistry. 2019;67:190-200. Epub 2019/04/06. doi: 10.1016/j.jnutbio.2019.02.012. PubMed PMID: 30951973.

33. Vehmanen L, Saarto T, Blomqvist C, Taskinen MR, Elomaa I. Tamoxifen treatment reverses the adverse effects of chemotherapy-induced ovarian failure on serum lipids. British journal of cancer. 2004;91(3):476-81. Epub 2004/07/22. doi: 10.1038/sj.bjc.6601979. PubMed PMID: 15266329; PubMed Central PMCID: PMCPMC2409844.

**Supporting information**

**S1 Fig. Diagram of included and excluded participants in this lipid analysis.**

**S1 File. Minimal anonymized data set.**

**S2 File. A manuscript showing changes using track changes.**
