# Supplementary material for: Changes in lipid profiles during and after (neo)adjuvant chemotherapy in women with early-stage breast cancer: A retrospective study
Source: PLoS One. 2019 Aug 29;14(8):e0221866. doi: 10.1371/journal.pone.0221866 (PMC6715243; doi:10.1371/journal.pone.0221866)
Supplement: S3 File — (PDF) [file pone.0221866.s004.pdf]

AJE AMERICAN JOURNAL EXPERTS

EDITORIAL CERTIFICATE

This document certifies that the manuscript listed below was edited for proper English language, grammar, punctuation, spelling, and overall style by one or more of the highly qualified native English speaking editors at American Journal Experts.

Manuscript title:

Changes in lipid profiles during and after (neo)adjuvant chemotherapy in women with early-stage breast cancer: a retrospective study

Authors:

Wei Tian, Yihan Yao, Guocai Fan, Yunxiang Zhou, Miaowei Wu, Dong Xu, Yongchuan Deng

Date Issued:

May 8, 2019

Certificate Verification Key:

F5A5-9A31-5659-3BA7-FFB8

This certificate may be verified at www.aje.com/certificate. This document certifies that the manuscript listed above was edited for proper English language, grammar, punctuation, spelling, and overall style by one or more of the highly qualified native English speaking editors at American Journal Experts. Neither the research content nor the authors' intentions were altered in any way during the editing process. Documents receiving this certification should be English-ready for publication; however, the author has the ability to accept or reject our suggestions and changes. To verify the final AJE edited version, please visit our verification page. If you have any questions or concerns about this edited document, please contact American Journal Experts at support@aje.com.

American Journal Experts provides a range of editing, translation and manuscript services for researchers and publishers around the world. Our top-quality PhD editors are all native English speakers from America's top universities. Our editors come from nearly every research field and possess the highest qualifications to edit research manuscripts written by non-native English speakers. For more information about our company, services and partner discounts, please visit www.aje.com.
